# Cytotoxic T Cell-Derived Granzyme B Is Increased in Severe *Plasmodium Falciparum* Malaria

**DOI:** 10.3389/fimmu.2019.02917

**Published:** 2019-12-11

**Authors:** Lea-Christina Kaminski, Mathias Riehn, Annemieke Abel, Christiane Steeg, Denis Dekugmen Yar, Otchere Addai-Mensah, Francis Aminkiah, Ellis Owusu Dabo, Thomas Jacobs, Maria Sophia Mackroth

**Affiliations:** ^1^Protozoa Immunology, Bernhard Nocht Institute for Tropical Medicine, Hamburg, Germany; ^2^Kumasi Centre for Collaborative Research in Tropical Medicine, Kumasi, Ghana; ^3^Department of Medical Laboratory Technology, Faculty of Allied Health Sciences, Kwame Nkrumah University of Science and Technology, Kumasi, Ghana; ^4^Divisions of Tropical Medicine and Infectious Diseases, I. Medical Department, University Medical Centre Hamburg Eppendorf, Hamburg, Germany

**Keywords:** granzyme B, CD8^+^ T cells, malaria, severe malaria, *Plasmodium falciparum*

## Abstract

In *Plasmodium falciparum* malaria, CD8^+^ T cells play a double-edged role. Liver-stage specific CD8^+^ T cells can confer protection, as has been shown in several vaccine studies. Blood-stage specific CD8^+^ T cells, on the other hand, contribute to the development of cerebral malaria in murine models of malaria. The role of CD8^+^ T cells in humans during the blood-stage of *P. falciparum* remains unclear. As part of a cross-sectional malaria study in Ghana, granzyme B levels and CD8^+^ T cells phenotypes were compared in the peripheral blood of children with complicated malaria, uncomplicated malaria, afebrile but asymptomatically infected children and non-infected children. Granzyme B levels in the plasma were significantly higher in children with febrile malaria than in afebrile children. CD8^+^ T cells were the main T cell subset expressing granzyme B. The proportion of granzyme B^+^ CD8^+^ T cells was significantly higher in children with complicated malaria than in uncomplicated malaria, whereas the activation marker CD38 on CD8^+^ T cells showed similar expression levels. This suggests a pathogenic role of cytotoxic CD8^+^ T cells in the development of malaria complications in humans.

## Introduction

*Plasmodium falciparum* (*P. falciparum*) malaria leads to the activation of CD8^+^ T cells during the liver- as well as the blood-stage of the disease. Hepatocytes, the host cells for *plasmodia* in the liver-stage, express MHC-class-I and can be recognized by CD8^+^ T cells having a potential protective capacity. Several vaccine strategies, based on the circumsporozoite protein (CSP), a protein expressed on sporozoites in the early liver-stage or whole sporozoite-based vaccines, have employed the induction of CD8^+^ T cells against liver-stage antigens in murine models (1–4) as well as human vaccine studies (5, 6).

The role of CD8^+^ T cells during the blood-stage of *P. falciparum* remains yet ill-defined, although an increased activation of CD8^+^ T cells has been documented in humans (7). Most data on CD8^+^ T cells in blood-stage malaria are derived from murine models addressing their function in experimental cerebral malaria (ECM) using *P. berghei* ANKA infection of C57BL/6 mice (8). In murine malaria it was clearly shown that CD8^+^ T cells sequester in the brain and mediate endothelial leakage in a granzyme B (GrzB) and perforin-dependent cytolytic reaction (9–12).

In contrast to the pathogenic role of CD8^+^ T cells in ECM, their contribution to human blood stage malaria remains controversial. At least indirect evidence exist that they may play a role in severe malaria by contributing to the induction of anemia (13). Moreover, soluble T-cell activation marker, as well as neutrophil and monocyte activation marker in the blood of malaria patients, could be linked to disease severity (14).

In *P. falciparum* malaria, numerous attempts were undertaken to correlate the T cell phenotype and or cytokine production with the clinical outcome of the disease. Several studies indicate that the ratio of pro-inflammatory TNFα and anti-inflammatory IL-10 may influence disease outcome (15). Although this might represent a reasonable explanation for disease manifestation this dichotomy is not present in every cohort studied.

In our study we aimed to further investigate a potential contribution of CD8^+^ T cells in the development of malaria complications. By analyzing soluble T cell-derived mediators in plasma from Ghanaian children suffering either from uncomplicated malaria or severe malaria symptoms, we found an increase in GrzB levels when compared to healthy or asymptomatic children. We employed Hierarchical Stochastic Neighbor Embedding (HSNE) (16) to use an unbiased approach for identifying the main source of GrzB. CD8^+^ T cells were confirmed as the main T cell subset expressing GrzB. Children suffering from severe malaria showed an increased population of CD8^+^GrzB^+^ T cells in peripheral blood when compared to children with uncomplicated malaria, indicating a potential pathogenic role of GrzB-producing CD8^+^ T cells in malaria.

## Materials and Methods

### Study Population

Blood samples were collected as part of a cross-sectional study between June and August 2015 in the Bosomtwi District, Ashanti Region, Ghana. A detailed description of the study participants, further inclusion and exclusion criteria, and study procedures have previously been published (17). In summary, samples were collected from children belonging to four different groups: (1) Healthy children, (2) asymptomatically infected children, (3) children with uncomplicated malaria, and (4) children with severe malaria.

Samples of healthy (*n* = 41) and asymptomatic (*n* = 41) children between the ages of 5–13 years of age were collected at Jachie D/A Primary school. Healthy children (healthy donor = HD) were defined as afebrile and negative for Malaria as detected by a HRP2-based rapid diagnostic test. Asymptomatic children (AS) were afebrile but positive for *P. falciparum* by HRP2. Blood samples from children with malaria (1–12 years of age) were collected at the St. Michael's Hospital, Pramso, Ghana. Children with uncomplicated malaria (*n* = 32) were treated with oral artemisinin combination drug as outpatients (OP). Children with clinically diagnosed severe malaria (*n* = 34) were treated with intravenous Artesunate as inpatients (IP).

The children in the HD group were 8.5 years of age, children in the AS group were on average 9.1 years of age. The children in the two groups with acute malaria were on average younger. Children in the OP group were on average 5.7 years of age, children in the IP group were 4.7 years of age.

All children with acute, symptomatic malaria and 15 of 41 of the asymptomatically infected children were microscopically positive for *P. falciparum* infection by thin blood smear. The children treated as inpatients for severe malaria showed the highest parasitemia with a mean parasitemia of 4.5% (range 0.3–17.3%) whereas children with uncomplicated malaria had a mean parasitemia of 1.4% (range 0.1–5.7%). Asymptomatically infected children showed only low parasitemia <1% (*n* = 15) or no microscopically detectable parasitemia by thin blood smear (*n* = 26). Individual parasitemia levels as well as additional information on the study participants have been described before (17).

Blood was drawn into heparinized tubes (1–2 mL) before initiation of treatment or directly at the school and processed within 5 h after collection for *ex vivo* flow cytometry staining.

### Detection of *Plasmodium falciparum* Infection

Present *P. falciparum* infections were determined using CareStart™ Malaria HRP2 (PF) rapid diagnostic test. Implementation was carried out according to the manufacturer's protocol. Parasitemia was determined with thin blood smears stained with 4% Giemsa and examined under oil immersion (original magnification × 100).

### T Cell Analysis by Flow Cytometric Assay

All antibodies were purchased from BioLegend. The surface immunostaining was performed by adding anti-PD-1 (PerCP/Cy5.5, clone EH12.2H7), anti-CD8 (AF700, clone RPA-T8), anti-CD4 (BV510, clone OKT4), anti-CD69 (FITC, clone FN50), anti-CD38 (PE, clone HB-7) and anti-CD39 antibodies (BV421, clone A1) to 100 μl of whole blood. After incubation for 30 min at 4°C samples were lysed and fixed with RBC lysis/fixation solution (BioLegend) for 15 min at room temperature and washed with cold PBS. Afterwards samples were stained intracellularly by adding anti-CD3 (APC/Cy7, clone HIT3a), anti-FoxP3 (AF647, clone 259D), and anti-Granzyme B antibodies (AF647, clone GB11) using the Foxp3 transcription factor staining buffer (eBioscience) according to the manufacturer's protocol. The samples were analyzed using a BD LSR II flow cytometer. If possible, at least 100,000 cells were acquired in the lymphocyte gate. The flow cytometry data analysis was conducted with FlowJo for Mac, version 10.5.0 (FlowJo LLC, Ashland, OR, USA). All acquired cells were gated based on FSC/SSC for lymphocytes. Lymphocytes were further gated for single cells and subsequently for CD3^+^ and CD3^−^ cells. CD3^+^ cells were then gated for CD8^+^, CD4^+^, and CD4^−^CD8^−^ T cells. Fluorescence minus one (FMO) controls were used to set gates for granzyme B, CD38, and PD-1. The gating strategy and exemplary flow cytometry staining can be seen in Supplementary Figure 1.

### Data Processing and HSNE by Cytosplore

Data were cleaned of doublets in FlowJo. CD3^+^ lymphocytes of all patients were concatenated group wise (HD *n* = 41; AS *n* = 41; OP *n* = 36; IP *n* = 32) and exported as FCS-file. For further analysis, FCS-files were imported to Cytosplore version 2.2.0 and arcsin transformation with the factor of *150* were applied. The cell number of all concatenated groups were downsampled to 10^6^ cells/group. Hierarchical stochastic neighbor embedding (HSNE) analysis of all groups based on the expression of CD8, CD4, Granzyme B, CD38, CD69, and PD-1 were computed. The algorithm for HSNE and the processing in the Cytosplore software was described before (16, 18). Advanced parameters of Cytosplore were used as suggested (*RNG Seed* of *-1*; *#Checks in AKNN* of *512*; *#RW for influence* of *100*; *#RW for Monte Carlo* of *15; Random walks threshold* of *1,5*; *Random walks length* of *15*; *Pruning threshold* of *0*; *Mean Shift Gridsize* of *256*.) The number of Scales were set to 3. Landmark development and clustering were complete data-driven. Cluster Scale was set to *27* with the *#Iters* of *1000*; *Exag* of *125*; *ExpDec* of *50* and *Cluster Res* of *256*. Landmark-based clusters were used for heat map generation.

### Detection of GrzB in Plasma

Levels of GrzB in plasma were determined using LEGEND MAX™ Human Granzyme B ELISA Kit (BioLegend) with the application of 50 μl of plasma per well, according to the manufacturer's protocol.

### Statistics

The statistical analysis was performed using Kruskal–Wallis tests with Dunn's post-testing in Graph Pad Prism 7.

### Study Approval

Ethical approval was obtained from the Committee on Human Research, Publication and Ethics, School of Medical Sciences/Komfo Anokye Teaching Hospital, Kwame Nkrumah University of Sciences and Technology, Kumasi, Ghana on the 27th of November 2014 under the reference number CHRPE/AP/377/4. Written informed consent was given from legal guardians/parents of all participants prior to inclusion in the study.

## Results

### Increased GrzB Plasma Levels in Severe Malaria

**Figure 1:**
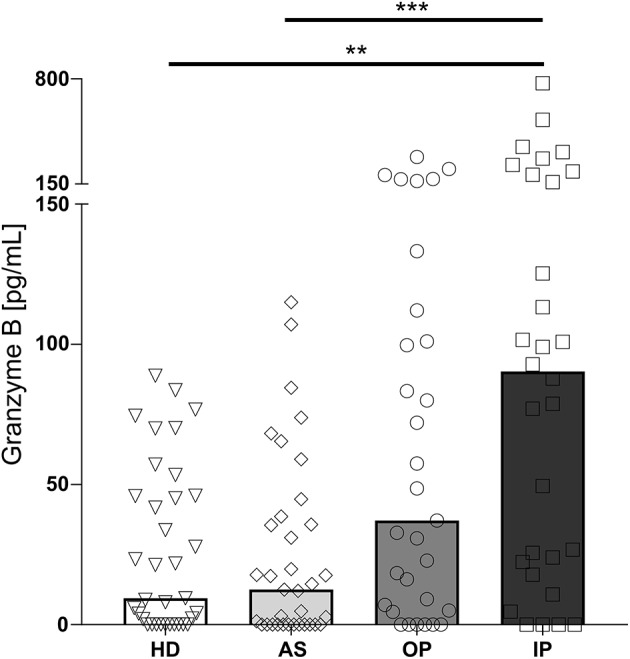
Granzyme B level in the plasma of *P. falciparum* infected children is associated with disease severity. Blood samples were taken from healthy, non-infected children (healthy donors, HD; *n* = 41) and asymptomatically infected children (AS, asymptomatically infected; *n* = 41) as well as from children with uncomplicated malaria (outpatients, OP; *n* = 35) and severe malaria (inpatients, IP; *n* = 32). Granzyme B levels in the plasma were measured by ELISA. Statistical significance was determined using Kruskal–Wallis Test with *post-hoc* Dunn's test: ****p < 0.0001; ***p < 0.001; **p < 0.01; *p < 0.05.

GrzB levels in plasma are reflecting a soluble marker for a recent activation of cytotoxic cells. Whereas, in plasma of healthy, non-infected children (HD) and asymptomatically infected children (AS) similar levels of GrzB were measured, children with uncomplicated malaria, treated as outpatients (OP) displayed a moderate increase, which did not reach statistical significance. In contrast, samples from children with severe malaria, treated as inpatients (IP), showed significantly increased levels compared to the afebrile children and healthy donors (Figure 1).

### Increased GrzB Production by CD8^+^ T Cells in Severe Malaria

**Figure 2:**
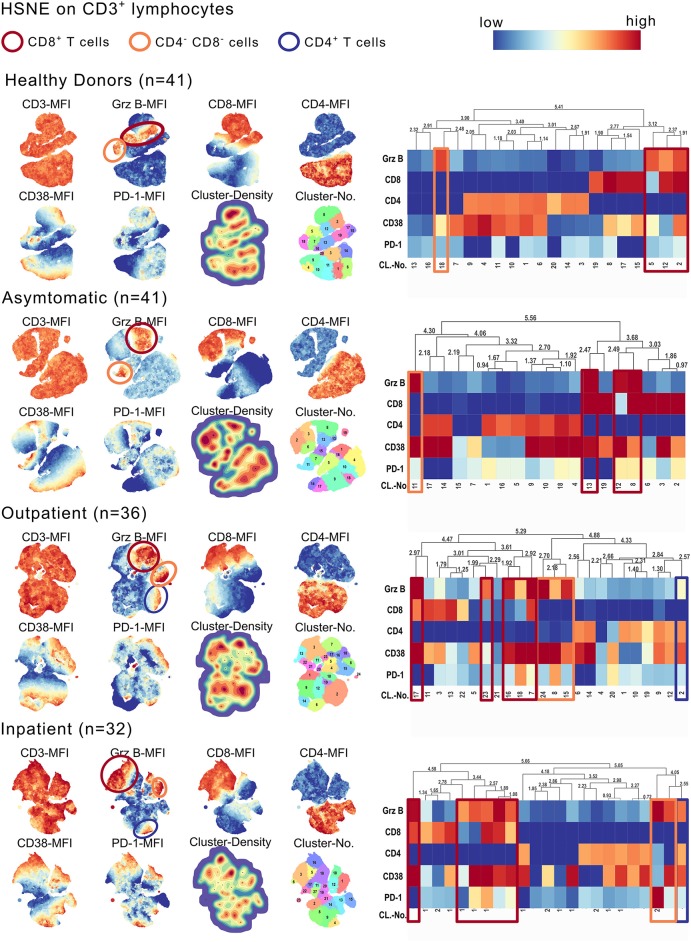
CD3^+^CD8^+^ T cells express high amounts of GrzB in *P. falciparum* infected children. CD3^+^ lymphocytes of all study participants were concatenated group wise (HD *n* = 41; AS *n* = 41; OP *n* = 36; IP *n* = 32). The cell number of all concatenated groups were downsampled to 10^6^ cells/group. Hierarchical stochastic neighbor embedding (HSNE) analysis of all groups based on the expression of CD3, CD8, CD4, Granzyme B, CD38, and PD-1 were computed. Landmark development and clustering were complete data-driven. Landmark-based clusters were used for heat map generation. Color code represents mean fluorescence intensity (red = high; blue = low). Blue boxes indicate GrzB-producing CD4^+^ T cell subsets, red boxes indicate GrzB-producing CD8^+^ T cells. Orange boxes highlight CD3^+^CD4^−^CD8^−^ T cell subsets, producing GrzB.

With hierarchical stochastic neighbor embedding (HSNE), we chose an unbiased approach to identify the source of the GrzB in the CD3^+^ T cell subsets. HSNE calculates the similarity of cells and forms cluster, reflecting distinct cell subsets. Besides a moderate fraction of CD4^−^CD8^−^ T cells and a minor fraction of CD4^+^ T cells producing GrzB, we could identify CD8^+^ T cells as being the main GrzB producing T cell subset in malaria (Figure 2). The number of GrzB positive CD3^+^ T cell subsets increased with more severe symptoms of malaria (Figure 2). One GrzB producing CD4^+^ T cell subset was detectable in OP and IP groups, but none in healthy and asymptomatic individuals (blue boxes in Figure 2). Two CD3^+^CD4^−^CD8^−^ T cell subsets were producing GrzB in the inpatient group, but only one in the healthy and asymptomatically infected groups (orange boxes in the heatmaps of Figure 2). However, already three T cell subsets, positive for CD8, produced GrzB in healthy controls. This increased to five distinct GrzB^+^CD8^+^ T cell subsets in the OP group and six in the IP group (red boxes in the heatmaps of Figure 2), indicating an increase of number and diversity of CD8^+^ T cells positive for GrzB.

**Figure 3:**
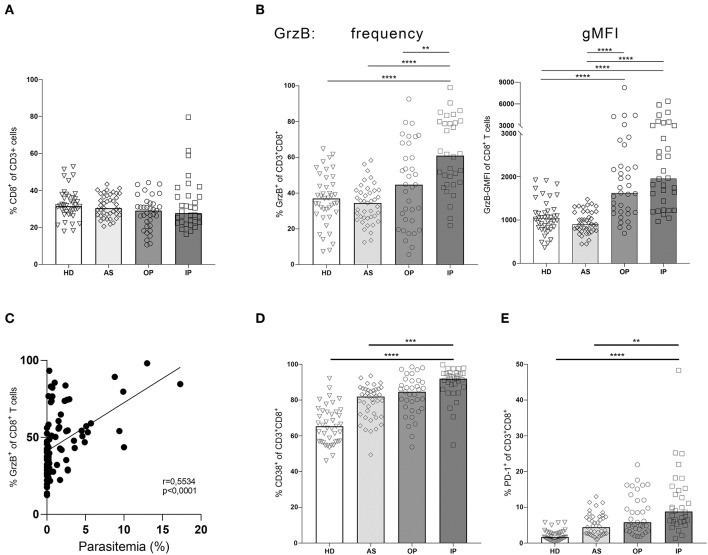
Granzyme B expression in CD8^+^ T cells correlates with disease progression. Blood samples from children infected with *P. falciparum* but lacking symptoms (AS, *n* = 41), children treated as outpatients for uncomplicated malaria (OP, *n* = 35), children treated as inpatients for severe malaria (IP, *n* = 32), as well healthy, non-infected children (HD, *n* = 41) were stained with fluorescent labeled αCD3, αCD8, αCD4, αGranzyme B, αCD38, and αPD-1 antibodies for flow cytometric analyses. **(A)** Frequencies of CD8^+^ T cells among CD3^+^ cells were assessed in all 4 groups. **(B)** The CD8^+^CD3^+^ T cells were further analyzed for the expression of the effector molecule granzyme B, **(D)** CD38 and **(E)** PD-1 in all four groups. Statistical significance was determined using Kruskal–Wallis Test with *post-hoc* Dunn's test: ****p < 0.0001; ***p < 0.001; **p < 0.01; *p < 0.05. **(C)** The correlation between the frequency of GrzB expression by CD3^+^CD8^+^ T cells and parasitemia was analyzed in the blood of all infected children (*n* = 89), using Spearman rank correlation.

To directly compare the frequency of specific cell subsets and the expression levels of GrzB, we used a classical gating strategy (see Supplementary Figure 1). Importantly, we could not detect differences in the frequency of CD8^+^ T cells contributing to the total CD3^+^ T cells in all groups (Figure 3A). Comparing the frequency of GrzB^+^ among CD8^+^ T cells within the 4 groups, the OP group showed a moderate increase of CD3^+^CD8^+^GrzB^+^ T cells without reaching statistical significance in comparison to healthy controls (HD) or asymptomatic children (AS) (Figure 3B). In contrast, a strong and significant increase of the frequency of CD3^+^CD8^+^GrzB^+^ T cells was found in the IP group when compared to all three other groups (HD = 36.48% ± 14.4; AS = 33.79% ± 10.90; OP = 44.47% ± 23.25; IP = 61.58 % ± 20.35). The HD and the AS group displayed similar fractions of CD3^+^CD8^+^GrzB^+^ T cells. Similarly, the overall GrzB content of the CD8^+^ T cell subset increased with disease severity, with OP and IP groups showing higher geometric mean fluorescent intensities of granzyme B (gMFI) (Figure 3B), compared to healthy and asymptomatically infected children. These results indicate that the CD8^+^ T cell subset of IP children produced more GrzB than those of HD, AS and OP groups. Interestingly, the frequency of GrzB expression by CD8^+^ T cells also correlated with the parasitemia (Figure 3C, *r* = 0.5534, *p* < 0.0001).

By further analyzing the induction of the activation marker CD38 on CD8^+^ T cells we could show a significant higher expression in the IP group compared to the HD and AS group but just a slight and not significant increase compared to the OP group (Figure 3D). Interestingly, a similar expression pattern was observed for the exhaustion marker PD-1 on CD8^+^ T cells. CD3^+^CD8^+^PD1^+^ T cells were increased in the IP group compared to HD and AS but not to OP (Figure 3E), indicating that PD1 expression might reflect the activation status of CD8^+^ T cells, rather than exhaustion in malaria.

Additionally, we compared the frequencies of GrzB^+^CD3^−^ cells and GrzB^+^CD3^+^CD4^−^CD8^−^ T cells between healthy controls, asymptomatically infected children as well as children with uncomplicated (OP) and severe malaria (IP) (Supplementary Figure 2). Of note, specific markers for unconventional T cell subsets or NK cells had not been included in our flow cytometry analyses. The frequencies of GrzB^+^CD3^−^ cells did not differ between all 4 groups (Supplementary Figure 2A). CD3^+^CD4^−^CD8^−^ T cells showed a higher percentage of GrzB^+^ cells in IP children compared to healthy and asymptomatically infected children but no significant difference was detected between the frequency of GrzB^+^CD3^+^CD4^−^CD8^−^ T cells in OP and IP children (Supplementary Figure 2B). Of note, the induction and role of GrzB producing CD4^+^ T cells in malaria has already been analyzed and discussed before (17).

## Discussion

In the present study we found increased plasma levels of GrzB in children with acute malaria in comparison with healthy children and children with asymptomatic malaria.

GrzB is produced by cytotoxic cells like NK cells (19) and CD8^+^ T cells (20). In various inflammatory diseases GrzB spills over from direct contact between cytotoxic cells and target cells and can be measured in the circulation, which might represent a surrogate marker of immune activation during infections (21) or autoimmune disease (22).

However, soluble GrzB failed to discriminate between children with uncomplicated and severe malaria in our study. Several factors might influence GrzB concentration in the circulation like half-life in plasma and binding to various matrix proteins. In addition, no data on the onset of disease is available in the present study and soluble GrzB might change over time. Therefore, the cellular source of GrzB was further investigated.

Using an unbiased approach based on flow cytometry data, we identified CD8^+^ T cells as the major T cell producers of GrzB in our patients and showed a strong expansion of GrzB^+^CD8^+^ T cells in children with acute malaria compared to afebrile children. Importantly, the proportion of GrzB^+^CD3^+^CD8^+^ T cells allowed a discrimination between uncomplicated and severe malaria in our patients, suggesting a role of GrzB^+^CD8^+^ T cells in the pathogenesis of malaria complications.

So far, data from human studies on CD8^+^ T cells in blood stage malaria are limited. However, there is strong evidence that CD8^+^ T cells play a protective role in the immune response to several other parasitic infections in humans such as *Toxoplasma gondi* or *Trypanosoma cruzi*, in which CD8^+^ T cells are essential for controlling the infection in the chronic disease state (23, 24). CD8^+^ T cells directed against pre-erythrocytic antigens of plasmodia can prevent the progression from liver stage to blood stage malaria and provide sterile immunity in human and murine malaria vaccine studies (25–28).

Yet in the blood stage of *P. falciparum*, CD8^+^ T cells were thought to play a minor role as the activation of CD8^+^ T cells depends on the expression of antigens via MHC I and red blood cells lack MHC I expression. In murine malaria models, however, it could be demonstrated that parasite-specific CD8^+^ T cells are induced during blood-stage malaria, probably through cross presentation of parasite antigens by CD8α^+^ dendritic cells or stroma cells (29) or even endothelial cells (30) and contribute to the development of experimental cerebral malaria (9, 29, 31). In humans, at least indirect evidence of a pathologic role of CD8^+^ T cells was given before by a study from Nigeria, in which high plasma sCD8 levels as surrogate marker for activated CD8^+^ T cells were associated with the development of severe malaria anemia (13). Our study now shows for the first time a potential link between the expansion of cytotoxic CD8^+^ T cells and severe malaria in pediatric patients. Interestingly, the expression of the activation marker CD38 and the immune checkpoint regulator PD-1 was not different between children with uncomplicated or severe malaria in our study. This supports the idea that not only the mere number of activated CD8^+^ T cells but a difference in their function, namely GrzB production, might be causally linked to severe symptoms. Several studies using experimental cerebral malaria models already demonstrated that CD8^+^ T cells contribute to endothelial dysfunction in a perforin (32) and GrzB-dependent mechanism (9). One could envisage a scenario in which endothelial cells present plasmodial antigens on MHC-I and are subsequently lysed by CD8^+^ T cells (8). Apart from a direct cytolysis GrzB was shown to modify cell function by its proteolytic activity (33).

Importantly, we found a positive correlation between CD3^+^CD8^+^GrzB^+^ T cells and parasitemia. This suggests that an increased antigen density influences the proportion of activated T cells producing GrzB. The strong GrzB production by CD8^+^ T cells, however fails to control or lower the parasitaemia in *P. falciparum* malaria in our patients. This is in contrast to *P. vivax*, where a direct and protective interaction between infected erythrocytes and CD8^+^ T cells has been demonstrated. This effect is attributed to the MHC-I expression of reticulocytes as host cells of *P. vivax*, which makes them susceptible for a granulysin-mediated permeabilization and subsequent GrzB-mediated killing (15).

Besides CD8^+^ T cells, other cell subsets such as NK cells and γ/δ T cells are important producers of granzyme B. However, our study focused on the role of CD8^+^ T cells in malaria and no specific markers for the analysis of NK cells or unconventional T cells or other cell groups such as monocytes or neutrophils were included, which is a limitation of the study. As surrogate markers, CD3^+^CD4^−^CD8^−^ T cells as well as CD3^−^ cells were analyzed for the proportion of GrzB^+^ cells but no significant difference could be detected between children with uncomplicated and severe malaria. Future studies are needed to further dissect a potential role of NK cells and unconventional T cell subsets to the development of malaria complications.

In summary, our data show that the expansion of GrzB^+^CD8^+^ T cells correlates with parasitemia and is associated with the development of complications in children with *P. falciparum* malaria, supporting prior observations from murine malaria models.

Further studies are needed to examine how GrzB contributes to the pathophysiology of severe malaria in humans.

## Data Availability Statement

The datasets generated for this study are available on request to the corresponding author.

## Ethics Statement

The studies involving human participants were reviewed and approved by the Committee on Human Research, Publication and Ethics, School of Medical Sciences/Komfo Anokye Teaching Hospital, Kwame Nkrumah University of Sciences and Technology, Kumasi, Ghana. Written informed consent to participate in this study was provided by the participants' legal guardian/next of kin.

## Author Contributions

TJ, EO, and MM designed the study. AA, CS, FA, and MM conducted the experiments. OA-M, DY, and EO recruited the patients and supervised the study in Ghana. LK and MR analyzed the data and prepared the figures. L-CK, MR, MM, and TJ wrote the manuscript. All authors reviewed the manuscript.

### Conflict of Interest

TJ, AA, MM, CS, and EO have filed a European patent application PCT/EP2017/080433 “Method for Diagnosing Different Forms of Malaria.” The remaining authors declare that the research was conducted in the absence of any commercial or financial relationships that could be construed as a potential conflict of interest.
