# Supplementary material for: Cytotoxic T Cell-Derived Granzyme B Is Increased in Severe Plasmodium Falciparum Malaria
Source: Front Immunol. 2019 Dec 11;10:2917. doi: 10.3389/fimmu.2019.02917 (PMC6918797; doi:10.3389/fimmu.2019.02917)
Supplement: Supplementary file 1 [file Data_Sheet_1.PDF]

Supplementary Figure 1: Gating Strategy and FMO controls

A

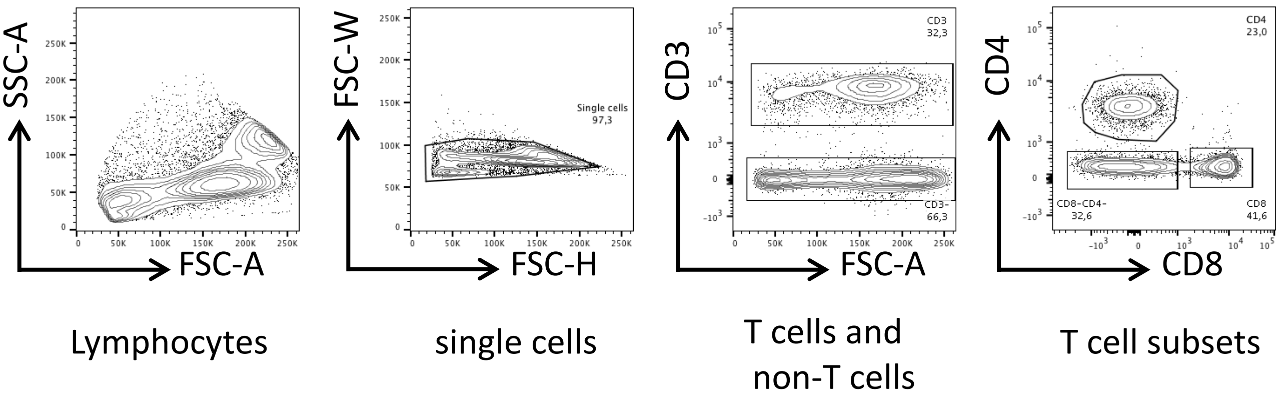

B

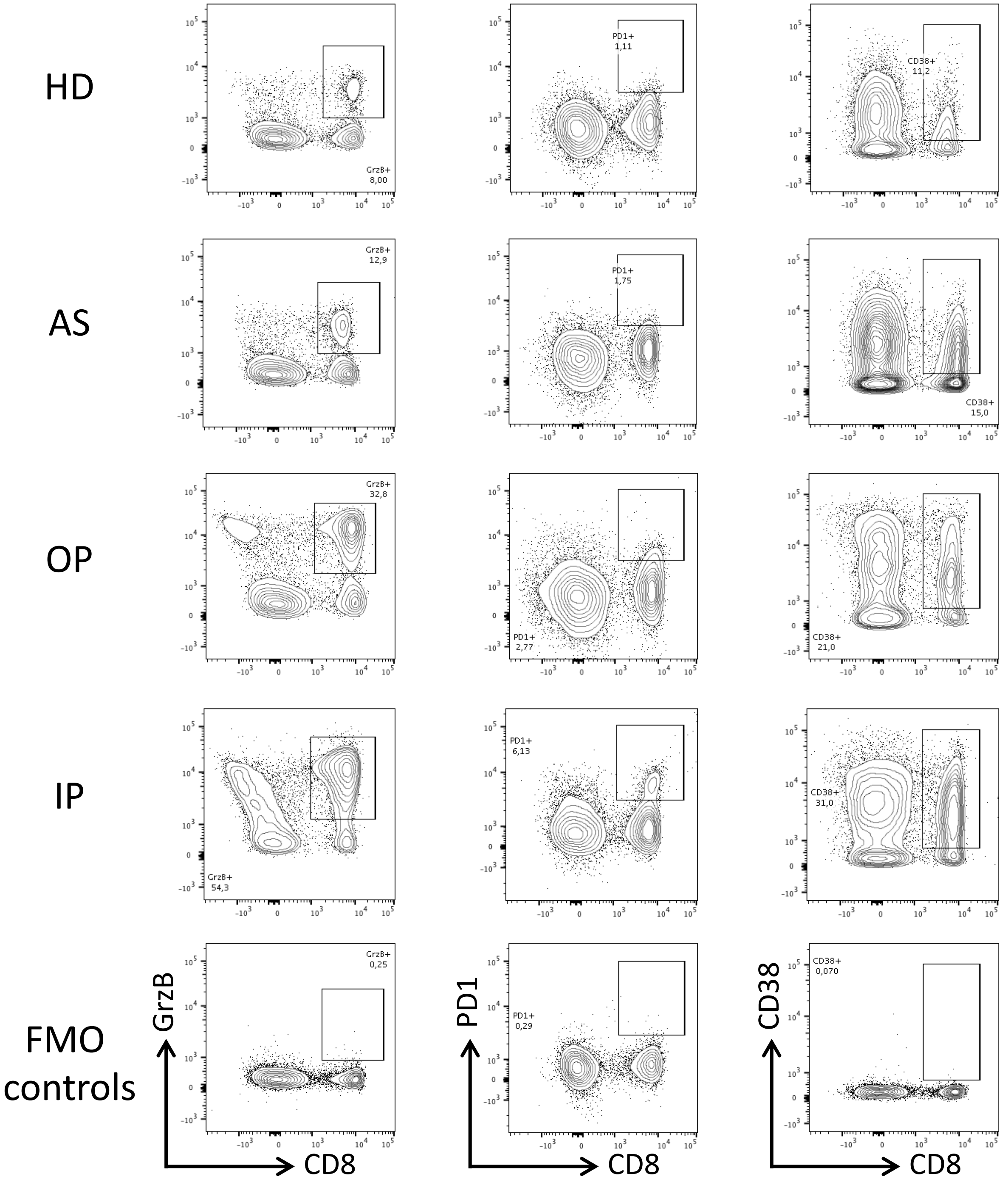

Supplementary figure 1: Gating strategy and exemplary stainings for PD-1, CD38 and Granzyme B
(A) Lymphocytes were gated for single cells, followed by CD3. $CD3^+$ T cells were further gated for $CD8^+$ and $CD4^+$ T cells and $CD3^+CD4^-CD8^-$ T cells. (B) Exemplary stainings for PD-1, CD38 and Granzyme B, based on fluorescence-minus-one (FMO) controls, are shown of each study group: healthy, non-infected children (HD), children infected with *P. falciparum* but lacking symptoms (AS), children treated as outpatients for uncomplicated malaria (OP) and children treated as inpatients for severe malaria (IP).

Supplementary Figure 2:
GrzB-expression of $CD3^-$ lymphocytes and $CD3^+CD4^-CD8^-$ T cells

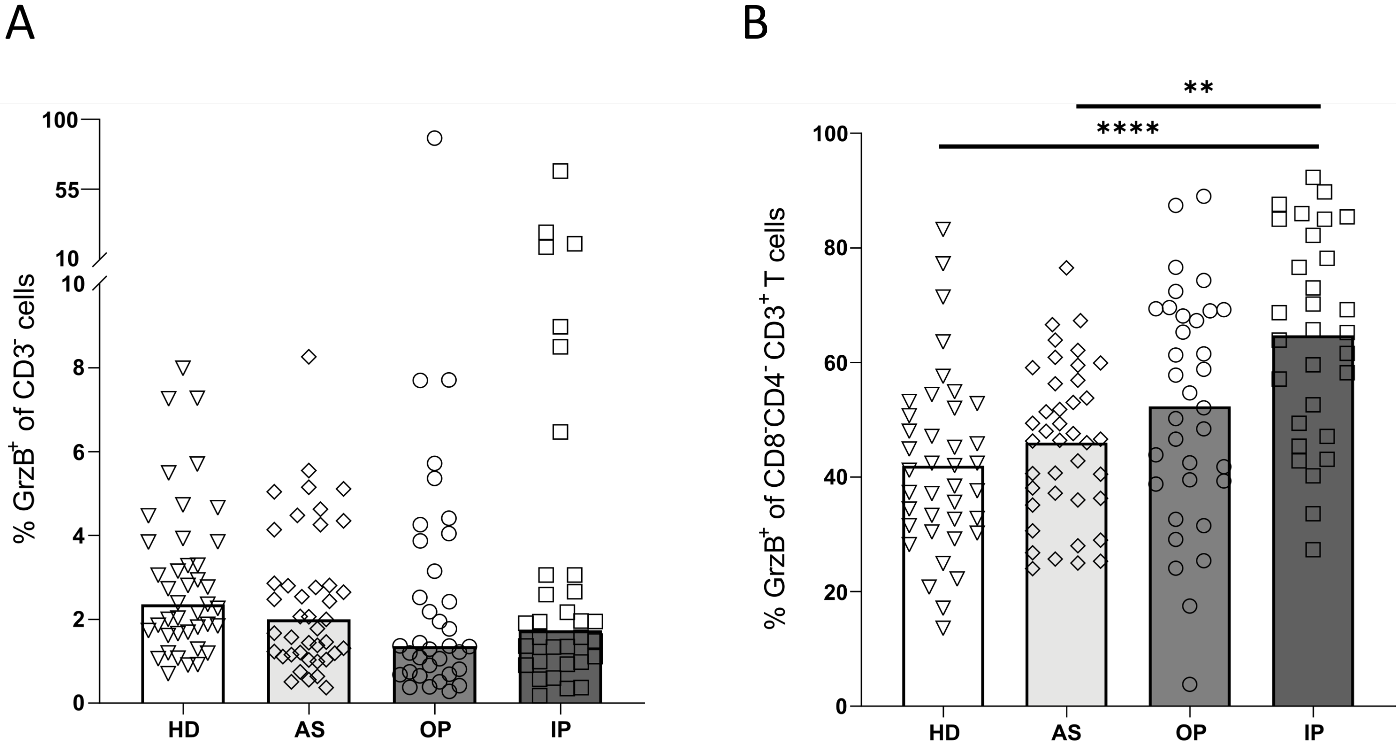

Supplementary figure 2: GrzB-expression of $CD3^-$ lymphocytes and $CD3^+CD4^-CD8^-$ T cells
The proportion of $GrzB^+$ cells was analyzed among CD3- lymphocytes (A) and the $CD3^+CD4^-CD8^-$ T cell subset (B) in all four study groups: healthy, noninfected children (HD, n=41), children infected with *P. falciparum* but lacking symptoms (AS, n=41), children treated as outpatients for uncomplicated malaria (OP, n= 35) as well as children treated as inpatients for severe malaria (IP, n= 32). Statistical significance was determined using Kruskal-Wallis Test with post-hoc Dunn´s test: ****= p<0.0001; ***= p<0.001; **= p<0.01; *= p<0.05.
